# The Effects of Cold upon the System

**Published:** 1873-01

**Authors:** R. W. Murphy

**Affiliations:** Sacramento, Cal.


*The Effects of Cold upon the System.* By R. W. Murphy, M.D., Sacramento, Cal.

As an underlying primary cause of disease, there is, perhaps, none wider in extent or greater in variety than cold. Its influence is as broad as the human race, reaching from infancy to old age; embracing alike in its deathly grasp the rich and the poor—the king and the beggar. No occupation, position, nationality or color can claim exemption; all are alike its subjects. The effects of cold upon the system, and the diseases and mortality consequent upon it, are truly alarming. Its victims outnumber those of cholera, small-pox and rum, all combined. Thousands, yea, millions, are being carried away annually by this agent. All classes, both of physicians and laity, seem, by a common consent, to admit its claims to and supremacy over mankind, without questioning its right, or offering any resistance to its secret invasion. The temperance orders keep a standing army in the field to try to keep down the rum traffic, and prevent its poisonous influence among mankind. We also have our quarantine laws, for protection against the introduction of cholera, small-pox, or any epidemic disease calculated to increase our mortality reports, or render our sanitary condition less secure. And when such epidemics are found among us, every effort is made by our boards of health to stay their onward spread; sanitary laws are strictly enforced, signals displayed, liberties restrained, and the various disinfectants used to keep down the enemy and drive him from our midst. While, upon the other hand, cold does not so much as receive a thought from any of our teachers of medicine, physiology or hygiene, unless they should be laboring under an attack of it at the time, although their attention is called to the subject almost daily; for you meet a friend, and how common the remark in connection with the compliments of the day, "All well, except a bad cold."

How often we hear the remark in the sick room, "Doctor, I have taken cold," or, "My child has taken cold;" and if the doctor is asked what is the cause of the sickness, he will doubtless, in a majority of cases, say it is from cold, especially if he is at a loss to comprehend the pathology of the disease. And the remark has become so proverbial, that the answer is quite satisfactory.

We have, as the result of this agent, a list of diseases that would well nigh exhaust the nosology of Cullen, and astonish the student of pathology. A classification of them would be too voluminous for this short essay. Suffice it to say that there is not a bone, nor muscle, nor tissue, in the system, but may have its physiological functions suspended, and become the seat of a pathological derangement, through the agency of cold. It should be regarded as the great enemy of physiology; as the father of pathology.

The external action of cold upon the system, when uniform, and continued for some time, produces general peripheral capillary contraction, depriving the parts of their usual amount of blood, thereby throwing a proportional amount upon the brain or some internal organ, causing hyperemia and congestion of the parts, with all the serious and fatal results we so often find. Professor Gross, in his remarks upon the primary effects of cold upon the general system, says: "They are those of an agreeable stimulant; the circulation is increased in force and frequency, a slight glow pervades the surface, and the individual is universally exhilarated. By and by this agreeable feeling is changed into one of pain and torpor; the brain is oppressed, as if under the influence of a powerful narcotic. A feeling of numbness and weight follows, with a peculiar prickling sensation." If the impression is maintained for any length of time, the parts become stiff and insensible, the blood, retreating from the surface, leaves it of a pale, whitish aspect, and gangrene and death soon follow.

Professor Gross further remarks that frost-bite was very prevalent among the English troops during their first winter in the Crimea, and the French suffered in still larger numbers, as well as more severely. On the 21st of January, 1855, not less than 2,500 cases of frost-bite were admitted into the French ambulances, and of these 800 died, death in many instances having, no doubt, been expedited by the effects of erysipelas, pyemia and hospital gangrene. Out of 460,000 soldiers lost by Napoleon in his Russian campaign, at least one-half died from the direct effects of cold upon the system. I think the disease from the direct action of cold is much greater than is generally estimated, but, considerable as it may be, it is small as compared with the reflex chemical action of this agent.

How do we take cold?

We do not take cold, in the general acceptation of the term, by merely getting cold, especially if the action is uniform over the system. This is well illustrated in the use of the Turkish baths, when a person coming out of a room with a temperature of 100 or 110, and while in a high state of perspiration, is showered with cold water without any bad results.

The frequent falling into the water when cold as ice, or, in some instances, breaking the ice to get to the water to immerse a subject, with no bad results, are additional evidences of the fact. But let one of those same persons get their feet wet, or a current of cold air blow across them when in a state of perspiration, or recline a few seconds upon the damp ground, or against a damp, cold wall, and the results are, they have taken cold. Dr. Austin Flint, in the third edition of his "Practice," says, in his remarks upon bronchitis, that the "disease is frequently, if not generally, produced by the action of cold." Exposure to cold is supposed to produce this disease by interrupting the eliminative functions of the skin, whereby an increased duty is thrown upon the pulmonary mucous membranes, thereby inducing congestion.

"This view of the causation is inconsistent with the fact, that a large proportion of cases of bronchitis are not traceable to any unusual exposure to cold. There is abundant evidence, however, to show that exposure of a portion of the body to a current of air is liable to excite an attack of this disease."

Another idea, very generally entertained, that the application of cold to a part of the system, depriving the part of its usual amount of blood by producing an increased capillary contraction, which causes an undue amount of blood to be thrown upon the weakest parts of the system, creating hyperemia and congestion of those parts, is also unsupported by facts. If this theory be correct, a person who is enfeebled from the loss of blood, whose volume has been reduced forty or fifty ounces, should not take cold as readily as the person whose system is full of blood, and every organ supplied with all it can contain consistent with the laws of health. But, do we not find the reverse to be true? Again, should this theory be correct, the catamenial flow should be increased, instead of suppressed or suspended, from the action of cold, since the discharge is found to be identical with the blood, and the uterus to be in as highly an impressible condition as is consistent with the laws of physiology. But our experience renders a different verdict. Local hemorrhages should be increased when the application of cold is to some other part of the system, instead of retarded or suppressed, as we often find to be the case when a person takes a bad cold. It is the reflex chemical action of cold upon the system that claims our attention at present.

It is now an established fact, according to Bernard, Brown-Sequard, and others, that the contractile elements of the blood-vessels are presided over by motor nerves. These are, in consequence, named vaso-motor nerves. Their action produces diminution in the caliber of the blood-vessels. If the action of the vaso-motor nerves be suspended, the blood-vessels dilate, most probably because their contractile elements being paralyzed, the pressure of the blood is able to distend the vessels. The vaso-motor nerves are derived immediately from the sympathetic ganglia. But Ludwig and Thiry have shown that the nerve-cells presiding over them are not contained in the sympathetic ganglia, but in the medulla oblongata. They locate this great vaso-motor center, in the experiments they made, to extend from one millimeter below the corpora quadrigemina to five millimeters above the calamus. It lies on either side, at a little distance from the middle line. From this center, the vaso-motor nerves pass down the spinal cord to all of the system. They leave the cord in the anterior roots of the spinal nerves; from thence they either pass through or receive branches from the ganglia upon the sympathetic and proceed to the blood-vessels.—*London Lancet*, of April, 1872. There are some small peripheral vaso-motor centers capable, no doubt, of producing a reflection of nerve-force, but to so limited an extent as not to claim our attention at present.

Professor Rutherford, of King's College, in the same article, says: "The general vaso-motor center in the medulla oblongata appears to be in almost constant action, thereby keeping up some degree of contraction of the vessels. The activity of the center may be increased or diminished by the excitement of various nerves. It may be increased by stimulating sensory nerves, by accumulation of carbonic acid in the blood, and by certain emotions. *Increase of its action* following the *stimulation* of *afferent nerves* leads us to think that there is a reflex-nerve mechanism whose nerve-center is in the medulla oblongata."

The action of cold upon the system *through* this great vaso-motor center is effected from all points through the afferent nerves. From the exceedingly fine peripheral nervous structure, the afferent nerves have their origin in fine nerve-tubes, which contain their protean threads or axis cylinder; these form into fine bundles, surrounded by a medullary sheath, which prevents the interruption of molecular motion from the other forces or motions in the system.

According to Herbert Spencer, "these fibres have their termini in a nerve vesicle or cell. The very delicate membrane that envelopes the nerve fibres becomes the inner lining of the nerve-cell vesicle, and is a non-conductor of molecular motion. The nerve vesicle not only forms a connecting link between the afferent, efferent and centripetal nerves, but it also becomes a reservoir of molecular motion, which is given out when disturbed, being composed of vesicular matter."

The lines of continuity from this vesicle, or primary nerve-center, to a higher one approximating the spinal cord, are called by some the centripetal nerves, but are identical with the afferent nerves, with an increased capacity for conveying molecular motion. Through these nerves and vesicles the peripheral disturbances reach the spinal cord, and through the cord, by similar nerves, up to the great vaso-motor center in the medulla oblongata, where the force is reflected through the vaso-motor nerves to any or all parts of the system. Dr. Dittman, in his experiments, says he thinks it is certain that there is a system of fibres in the substance of the spinal cord which, though they do not belong to nerve roots, are capable of responding to the action of direct stimuli, and can transmit the impressions thus generated along the whole length of the spinal cord up to the medulla oblongata, where they undergo reflection into motor nerves.—*London Lancet*, of May, 1872.

The fact that the general application of cold to all the system at once does not result in the person taking cold, is because the molecular action of all the afferent nerves is uniform; that each and all of the vaso-motor centers are alike stimulated, and their action, although greatly intensified, is uniform; and their action being uniform, we have the like uniformity in the contraction and dilatation of the vascular system, consequently the equilibrium of the vital forces remains undisturbed. But it is not so when only a portion of the system is exposed to the stimulating action of this agent. The force transmitted by chemical action to the great vaso-motor center when only a small portion of the afferent nerves is called into action, is not sufficient to stimulate the entire vaso-motor center to action; the result is, that the force transmitted and reflected only calls into action one or two of the vaso-motor nerves. The action of these nerves is greatly intensified, causing a corresponding contraction of the blood-vessels over which they preside, the action falling mostly upon the capillaries and small arteries, which become so contracted as to interrupt and greatly retard the circulation of the blood in the parts, and we have, as the result, congestion, diminished supply of oxygen, blood poisoning.

With this view of the action of cold upon the system, we can readily understand how it is that a "slight peripheral disturbance from cold, setting up molecular action in the afferent nerves, which wave of molecular action upon reaching the first nerve vesicle, disturbs the unstable vesicular matter; this gives off its latent force, which adds to the force already being sent through the afferent nerves. From this primary nerve-center or reservoir of molecular motion, we have an *increased* molecular action propagated along the centripetal nerves until it reaches the great vaso-motor center, where it undergoes reflection into some of the vaso-motor nerves," whose terminal action may be in the lungs, the throat, the liver, the kidneys, the heart, the pleura, the eye, or the ear, producing disease of any or all the organs and tissues in the system. When the terminal action falls upon the joints or muscles, rheumatism, ostitis and periostitis are in like manner produced.

We think the action of cold is manifest in the development of boils, abscesses and carbuncles. In these the chemical action is quite limited in extent, but the intensity is sufficient to cause congestion of the parts, which deprives the protoplasm in the tissue-cells of the supply of oxygen necessary to sustain its vital relation; death of the cells is the result. This effete matter, though not larger than a pin's head, sustains the relation of a foreign body, and, unless absorbed, must result in elimination by suppuration. It further manifests itself in the development of the various tumors, both malignant and non-malignant. This is most likely caused by a diminished supply of oxygen, which so alters the vital condition of the cells that their physiological functions are no longer sustained. This altered condition of the cells still retains a *morbid* vital relation with the power to assimilate or metamorphose cells of its own character; thus building up, as it were, an independent growth in the system.

Cold, as a chemical agent, is most potent, as we all know. This agent, acting upon the peripheral nerve structures, as before remarked, evolves, by chemical action, a force which is communicated by molecular action along the afferent nerves to the great vaso-motor center.

This, like any other force in nature, naturally follows the line of least resistance; hence, the vaso-motor nerves most impressible would receive the force thus transmitted and reflected. The impressibility of nerve depends upon its peculiar vitality, whereby the molecules act readily and with such uniformity as to transmit an isomeric wave uninterrupted, or with greater facility than its neighbor.

Another fact in connection with nerve action is, that the more frequently a wave of molecular action is propagated along a nerve, the more readily will the molecules act. A person taking cold to-day, which is reflected into the lungs or throat, will be more likely to receive the next impression along the same line of nerves, than in any other part of the system.

The vital condition of the nerves is constantly undergoing a change, for, like other tissues, these nerve molecules are giving off effete matter and receiving new material and vitality from the blood, and are liable to the vicissitudes and interruptions that other tissues are, being at times more susceptible to impressions than at others.

A person may have such a condition of the nervous system to-day that exposure to cold fails to excite molecular action in the afferent nerves; but, in less than twenty-four hours, may receive and transmit the same with most wonderful effects. It would seem that the vaso-motor nerves presiding over the uterus have their vitality elevated, and the molecules rendered more susceptible to impressions during the catamenial flow than at other periods, which accounts for the catamenial suppression from the chemical action of cold.

We should also bear in mind that the greater the distance traversed by the afferent nerves, and the greater the number of nerve-vesicles contained in the line, the greater will be the force transmitted to the medulla oblongata. And well might the poet, upon beholding this strange phenomenon of nature, say:

> " The feet with the head hold wedded intercourse,
> And all by moods and tides."

The sanitary condition of the atmosphere has much to do with the chemical action of cold upon the system. Niemeyer, in his Practice, in speaking of influenza, says "that in the year 1732 it appeared through Europe, from east to west, in the form of an epidemic; attacked at least one-half of the population—the disease being dangerous to children and old persons. Since then, it has appeared several times as an epidemic—in 1800, also in 1835, with like results." And this able expounder of medicine and pathology assigns no other cause for this disease than cold. We find it true, that here in California, when we have those heavy northerly blows that visit us annually, the electrical and sanitary condition of the atmosphere is greatly changed, and the complaint of having taken a cold is very common, if not an epidemic. I think it is safe to predict that the time is not very remote when chemists will furnish the means by which the sanitary condition of the atmosphere will be as easily read as the thermal condition is at present.

You may regard my prediction as being both premature and mythical, but an element so indispensable to human life has not received that attention at the hands of science that its importance demands.

Moisture is another great auxiliary to the chemical action of cold upon the system, by reducing the temperature to a lower standard; also, by softening the effete cuticle, and rendering the peripheral nerve-structure more susceptible to chemical action. Hence it is that persons putting on under garments a little damp, or getting a foot wet, are liable to take cold.

Habit, also, has much to do with our taking cold, as is shown when a person who has been accustomed to wear a necklace, even small, will often take cold upon removing it. The removing of flannels from children, or taking off their shoes and stockings and running barefoot upon the damp ground, will cause them, most likely, to take cold; but in a short time, if continued in, the cold fails to excite molecular action in the afferent nerves, and the child can run in the cold, even in the snow, barefoot, with impunity. The state of the mind has much to do with our taking cold.

An excited, determined state of the mind liberates and sets up an internal molecular action from the cerebral organs, which may prevent the wave of molecular action from the afferent nerves reaching the medulla oblongata. But let the mind be at rest, or the individual be asleep, and the same chemical action which had been stayed in its progress now meets with no resistance, and the force, though light, is felt in some part of our system, interrupting the harmonious laws of physiology. I do not propose to consider cold as a therapeutical agent at present; its claims as such, in the hands of the skillful physician, are worthy of our consideration.

What is it to take cold? It is a suppression or suspension of the respiration of the parts, caused by the reflex chemical action of cold depriving the parts of their normal supply of oxygen, and causing a retarded or suspended elimination of carbonic acid.

What are the results? Dr. J. C. Peters, of New York, says: "We know that muscular tissue has natural electric currents of its own, which are constantly passing in certain definite directions as determined by delicate galvanometers. The center of each muscle-molecule is held to be normally in a state of positive electricity, whilst its poles or sides next the other nearest molecules are negative, thus resembling a zinc center and copper poles when immersed in diluted acid. The normal muscle currents are derived from the molecular chemical changes constantly going on in the nutrition of living tissues, and are connected with the process of osmosis which takes place through the sarcolemma. Disturbances of these chemical osmotic processes cause irritability and irregular contractions of the muscles."—*New York Medical Record.*

With the authority just referred to, I think I am warranted in saying that every tissue in the system, as well as the muscles, have their vital or electrical currents, which are governed and kept up by a law of animal chemistry; that the oxygenized or vitalized protoplasm in the cells is the basis of all healthy physiological functions of the system; and the results of our taking cold are a diminished supply of oxygen to the tissue, which impoverishes the protoplasm in the cells, and is the underlying cause of a large proportion of diseases. "And as the different sounds that greet the auditory are but so many altered conditions of the atmosphere, and our different thoughts but so many altered conditions of the ethereal essence of our spiritual natures," so are the various diseases but so many altered conditions of the vital forces of the system.

What can be done to prevent persons from taking cold? Uniformity of temperature is the great desideratum, together with a thorough and uniform system of ventilation, and uniformity of habit.

